# Innovating editorial practices: academic publishers at work

**DOI:** 10.1186/s41073-020-00097-w

**Published:** 2020-08-05

**Authors:** Serge P. J. M. Horbach, Willem Halffman

**Affiliations:** 1 Faculty of Science, Radboud University Nijmegen, Institute for Science in Society, P.O. box 9010, 6500 GL Nijmegen, The Netherlands; 2 Centre for Science and Technology Studies (CWTS), Faculty of Social Sciences, Leiden University, Wassenaarseweg 62A, 2333 AL Leiden, The Netherlands

**Keywords:** Publishing, In situ interviews, Commercial publishers, Peer review, Editorial process, Innovation

## Abstract

**Background:**

Triggered by a series of controversies and diversifying expectations of editorial practices, several innovative peer review procedures and supporting technologies have been proposed. However, adoption of these new initiatives seems slow. This raises questions about the wider conditions for peer review change and about the considerations that inform decisions to innovate. We set out to study the structure of commercial publishers’ editorial process, to reveal how the benefits of peer review innovations are understood, and to describe the considerations that inform the implementation of innovations.

**Methods:**

We carried out field visits to the editorial office of two large academic publishers housing the editorial staff of several hundreds of journals, to study their editorial process, and interviewed editors not affiliated with large publishers. Field notes were transcribed and analysed using coding software.

**Results:**

At the publishers we analysed, the decision-making structure seems to show both clear patterns of hierarchy and layering of the different editorial practices. While information about new initiatives circulates widely, their implementation depends on assessment of stakeholder’s wishes, impact on reputation, efficiency and implementation costs, with final decisions left to managers at the top of the internal hierarchy. Main tensions arise between commercial and substantial arguments. The editorial process is closely connected to commercial practices of creating business value, and the very specific terms in which business value is understood, such as reputation considerations and the urge to increase efficiency. Journals independent of large commercial publishers tend to have less hierarchically structured processes, report more flexibility to implement innovations, and to a greater extent decouple commercial and editorial perspectives.

**Conclusion:**

Our study demonstrates that peer review innovations are partly to be understood in light of commercial considerations related to reputation, efficiency and implementations costs. These arguments extend beyond previously studied topics in publishing economics, including publishers’ choice for business or publication models and reach into the very heart of the editorial and peer review process.

## Background

Academic peer review plays a crucial role in many of research’s core processes, including grant and career reviews, but especially in the editorial assessment of papers by research journals. The journal peer review system and the editorial process in which it is embedded are gatekeepers for the dissemination of research findings, act as an internal self-regulating mechanism, and, by acting as a selection mechanism, play a key role in the academic reward system [1–3].

Following a series of scandals and controversies over the ability of peer review to guard research quality or integrity, several innovative peer review procedures and supporting technologies have been proposed by a host of enthusiastic innovators, each motivated by specific concerns over dominant approaches [4, 5]. These include the introduction of various software tools, such as text similarity or statistics scanners; procedures of blinding or disclosing actors’ identities; shifting timing of peer review in the publication process; and new criteria for accepting or rejecting manuscripts. Some of the suggested solutions even move in opposite directions, such as increased anonymity, moving from single-blind to double-blind review, versus increased openness, revealing author and reviewer identities [5, 6].

Despite the many suggestions and innovations that promise to improve the peer review system, adoption of these new initiatives seems slow [7, 8]. On a global scale, editorial procedures are rather stable and traditional ways of organising the editorial process still prevail, despite evidence of flaws in old practices and proposed advantages of new ones [9–11]. Implementation of novel review procedures seems to be restricted to specific niches (specialties, publishing platforms), with the exception of the implementation of text similarity software or ‘plagiarism scanners’ [8]. Slow adoption may be partly explained by a lack of systematic evidence of their effectiveness [12]. Nevertheless, given the fierce promotion by their advocates [13], it may seem strange that new review practices do not convince a wider set of journals.

This raises questions about the wider conditions for peer review and editorial change and about the considerations that inform decisions to innovate. Many of the newly suggested procedures claim to improve the quality of published research or the fairness of the review process, but these are not the only considerations informing journal policies. Claims about how novel review formats improve quality, transparency or scrutiny of the published record – and ultimately might benefit research in its endeavour to produce and disseminate knowledge – are omnipresent in discussions on peer review (e.g. [6, 9]). The advocates of peer review innovations assume that these are the features that will carry forward a transformation. While the discourse about these innovations is thus inspired by strong academic values, it does not account for the current practical conditions of running a research journal. From the perspective of fairness and quality alone, it remains unclear how other considerations, such as publishers’ motives or other stakeholders’ perspectives, may affect decisions to innovate the editorial process. In contrast, the perspectives and business considerations of publishers have been highlighted in discussions on publication models such as open access publishing, or the establishment of hybrid journals [14, 15], but whether they extend into the heart of editorial decision making remains unclear.

From the perspective of a Science and Technology Studies (STS) understanding of innovations, it is not so strange that innovations fail to convince users by arguments of superior ‘quality’ alone. Apart from their meaning and specific performance, innovations require integration in existing practices and wider socio-technical configurations, with active involvement of users in light of practical concerns and relations to other actors. In the case of journal peer review and journal’s editorial process, this comprises several user perspectives, including opinions about varying peer review procedures in research communities, the willingness of authors and reviewers to participate in innovative formats, but especially how peer review innovations relate to existing editorial practices and policies. Hence some of the currently proposed peer review innovations may be more than just marginal improvements, and are rather suggestions that require transformative change, affecting not only peer review, but also publishing strategies and economics. It may also go beyond the realm of individual publishers and actors, affecting systemic aspects such as reviewer recognition and incentive structures [16]. Understanding the appeal of review innovations therefore requires comprehension of the wider editorial practices in which they are to land.

In this study, we set out to research editorial practices to reveal how these might benefit from peer review innovation, and to describe the considerations that inform such decisions. We carried out field visits and in situ interviews at the editorial office of two large academic publishers to study the editorial process in close detail and better understand the day-to-day practices and concerns of their employees at all levels. In addition, we interviewed editors of journals that are not closely related to large publishers. Specifically, we were interested in understanding the considerations that inform editorial transformation, guided by questions such as: what does the process of transformation look like? Who makes decisions about such changes? And on what basis are changes made? We hence mainly focus on intended, deliberately planned instances of transformation.

## Methods

In this article, we study the practices involved in the editorial and peer review process of large, commercial, academic publishers. Following Schatzki [17] and Reckwitz [18], we understand practices simply as ‘a routinized type of behaviour’ and a temporally and spatially dispersed nexus of doings and sayings. We will understand individuals as the *carriers* of practices and hence ‘know-how, meanings and purposes’ are not taken to be personal attributes, but rather ‘elements and qualities of a practices in which the single individual participates’ [18].

Following Shove, Pantzar [19], we will distinguish three elements of a practice: materials, competence and meaning. There is now broad agreement in practice theory that things (material objects) should be treated as elements of practice. Competence refers to the know-how, background knowledge and understanding required to either perform or evaluate a performance of a practice (though there might be a difference between the skills required to do both tasks). Meaning refers to the social and symbolic significance of participation in a practice at any one moment [19]. Actors act upon these meanings, regardless of whether their beliefs are objectively ‘true’, and hence it is essential to understand how they interpret their practices.

In the setting of the peer review and editorial process, the material elements of a practice include manuscripts, the email and the electronic submission system, and digital tools such as plagiarism scanners or statistics scanners. The competences involved comprise an academic knowledge level expressed as an academic degree, expertise in the relevant subject area, know-how of the review and editorial procedures, familiarity of the editor with the reviewer, English language skills, legal expertise or ethical background knowledge. Last, the meaning constituting the practice may involve a sense of academic duty, a willingness to improve research, an appreciation for keeping up with the literature, a desire to stay anonymous, a desire to make money or create business value, a lack of time, or commitment to a journal, a research field, or a company.

Unpacking the notion of practice a little further, we may consider peer review and editorial practices as an example of what Schatzki calls complex ‘integrative’ practices [17], since they embrace ‘a set of hierarchically organized doings, sayings, tasks and projects’. Shove, Pantzar [19] use a somewhat different notion and speak of ‘complexes of practices’ of which peer reviewing may constitute an example (embracing the more mundane practices of reading, writing, judging, emailing, etc.). Complexes of practices are cases in which practices come to depend upon each other, either in terms of sequence, synchronization, proximity or necessary co-existence. In such cases, emergent characteristics of the complex of practice cannot be reduced to the individual practices of which it is composed.

### Transforming practices

An important aspect of our study is the analysis of how editorial practices may be transformed or, on the contrary, what keeps them stable. In previous work on transformation of practices, authors have distinguished between gradual and radical transformation of a practice. In the former, learning, carrying and sharing may lead to the capture, commitment and change of some practices and practitioners: the processes are transformative both of the practitioners involved and of the practices they reproduce. In the latter, practices ‘die’ and new practices are born as ‘changes in organization are vast or wholesale, or a practice’s projects and tasks are simply no longer carried out’ [20]. One of the explanations for the extinction of old (bundles or complexes of) practices is that they had too little internal rewards and were hence not valued for their own sake, but rather as an instrument to obtain something else [21]. Other explanations refer to a lack of symbolic or normative anchoring as well as a lack of connection with and dependence on other practices. In the current publishing landscape, with large scale shifts in publication models triggered by open science and open access initiatives [22], both gradual and more radical transformation in the editorial process may be expected, we will hence focus on both in our analysis. In particular, we set out to observe the introduction of new editorial practices or changes in old processes.

In short, practices die out when links between their constituting elements are no longer reproduced. This could for example happen when particular actors no longer have the resources, or competence to enact a certain practice. One could think of a journal no longer having access to specialist statistics reviewers. Similarly, bundles or complexes of practices discontinue when one of the practices constituting them disappears. This can either happen through materials not being available anymore (or changes in materials, such as modifications in the electronic submission system), competences disappearing, or shifting meanings. Our study examines which factors have most impact on shifting, disappearing and evolving editorial practices.

Besides focussing on what initiates transformation of practices, a fruitful lens may be to look at what keeps practices constant and facilitates reproduction over time. One of the factors important in keeping practices stable comprises the infrastructures in which practices are embedded [23]. These infrastructures allow for routinized actions and maintain the links between different (elements of) practices, thereby keeping them stable over space and time [24]. Monitoring and feedback play another essential role in the maintenance of practices. It helps them develop, adapt or stay constant over time, as well as help them travel through space and time. In this, it is useful to distinguish between forms of monitoring and feedback that link one instance of performance to the next, and those implicated in the unfolding careers of practices-as-entities. However, both forms critically interact and often connect. This may happen in at least three ways [19]:

- When the careers of individuals and practices intersect, monitoring may reveal important signs of progress and hence encourage further effort and investment of time and energy in future performances of a practice.
- Methods of measurement may end up changing the performances and the practices they are designed to monitor. Ample examples of this phenomenon have been described in the literature [25].
- Systems of classification and standards constitute ‘invisible mediators of action’ [26]. By setting these standards, templates are established by which performances are compared and which define what one enactment is a performance of.

Thus, technologies and instruments of feedback are of concrete relevance to establish and maintain circuits of reproduction, which in turn are of direct consequence for the survival and transformation of relations between practices and of practices (and their constituting elements) themselves.

In our previous work, we identified a large range of editorial procedures [5] and the current study examines how and why certain procedures are replaced by others from the perspective of practice rather than procedure. We hence conceptualise innovations of editorial procedures as mere changes from one system into another, in terms of our classification of editorial procedures, but studied from the perspective of the publishing practice.

### Methodological approach

Our findings are based on field visits (by SH) to two large, international, commercial academic publishers, both of which are not associated with universities and have a portfolio of several hundred journals and book series in a wide variety of disciplinary fields. As part of access negotiations, anonymity agreements were made, preventing us from providing more details about the publishers. In total, the field visits lasted for about 3 weeks and the collected material consists of field notes gathered during 41 interviews or individual meetings, and 10 group meetings. The group meetings usually lasted for roughly 1 h and were attended by four to over 20 people, with varying roles within the publisher (see section 4.1). Most meetings involved a virtual component with attendees from other offices attending through video connections. The interviews comprise mainly in situ conversations with members of the editorial offices, while they were at their usual working place, sometimes even while they were just carrying out their daily tasks. Sometimes, as not to disturb colleagues, a separate room was used for the interviews. All interviews were open interviews, with most questions naturally emerging from the conversations related to the interviewee’s daily practices and work environment, their experiences with changes in practices or procedures, and the context and rationale for such changes. Hence, the innovations discussed were mostly related to those that the interviewee was working with or directly related to. In addition, some innovations widely discussed in the academic literature, such as registered reports or open peer review, were sporadically put forward by the interviewer. With our work we follow the tradition of ethnographic fieldwork at publishers or publishing related organisations, for example by Hirschauer [27] and Jacob [28]. In addition, we conducted three interviews with (managing) editors of journals not closely related to such large publishers, mainly for contrast, aiming to get a sense of generalisability beyond large, commercial publishers. The editors were sampled through snowballing, being unknown to the authors but found through our personal networks. SH has previous experience in conducting qualitative interviews [29].

The first fieldwork visit took place at the editorial office of publisher A. At this publisher, we mainly focussed on a set of open access journals and the team managing and working for this set. The second fieldwork period involved the editorial offices of publisher B, holding a portfolio of both open access and subscription journals. At the offices of both publishers, SH observed and talked with staff responsible for multiple journals, in total spanning a portfolio of over 100 journals. Extensive field notes were taken during the interviews, meetings and the remainder of the fieldwork. In particular, the field visits comprised observing the daily and basic workflow of manuscript handling, as well as discussing the possibilities for changing this workflow. We refrained from making audio recordings for several reasons: In the open-plan style offices the quality of the recordings can often be poor, due to background noise. This means formal interviews can only take place in separate, quiet areas, requiring the interviewees to move away from their desk, where they would not be able to show what they are doing (but rather have to rely on explaining it verbally), and they are in a less familiar or comfortable place. Some interviews did take place away from the desk, but only at the interviewee’s suggestion, usually in order not to disturb their colleagues. In early conversations during the first field visit, we generally noticed that asking for recordings and having the recorder visible throughout the interview had an impact on the interviewees. They clearly tended to feel more reserved. Combining these considerations, we felt that we were able to collect richer and more accurate data when not recording the interviews. Alternatively, extensive notes were taken during the field visit, which were consequently processed and written out in full on the same day. Admittedly, this creates an extra layer of interpretation by the researcher and some information might have gone lost because of limitations in what could be written down during the conversations. Nonetheless, we felt that the benefits of not using recordings outweighed these potential drawbacks as it led to more natural, open conversations and discussions.

Data analysis followed traditional social science techniques for analysing qualitative research material: Following on data processing on the same day of data obtainment, SH further familiarised himself with the data (comprising of over 100 pages of field notes) through rereading and annotating it. Subsequently, all data were coded using the Atlas.ti software, using an inductive coding approach. Codes and common themes were discussed between both authors, after which some minor modifications of codes and some slight recoding were performed by SH. The main themes focussed on during the coding were those inspired by the research questions (editorial innovations, their context, rationale, and implementation), while some additional sub-themes emerged. These will be elaborated on in the next section.

In this article, data is anonymised to the extent that all names of publishers, journals and individuals are omitted. Informed consent to conduct the field trips and interviews was written, and actively given by the publishers’ management. The members of the offices visited were informed about this prior to the field visits. When presenting empirical data, we provide generic job titles or descriptions to contextualise quotes or data while protecting the anonymity of the individual. Representatives of the publishers involved, as well as all editors from the more or less standalone journals, read the manuscript prior to submission and were given the opportunity to comment on issues of anonymity and factual mistakes. They consent that the current manuscript is an accurate analysis of their statements.

## Results: innovating the editorial process

### Following a manuscript: the editorial process

In this section, we will first describe the practice of handling a manuscript by large publishers, following the editorial process from submission to the final decision of acceptance or rejection. Even though some differences exist in the way different publishers handle their manuscripts, the process is fairly similar in broad terms, at least among the large publishers.

#### Journals owned by the publisher

**Fig. 1:**
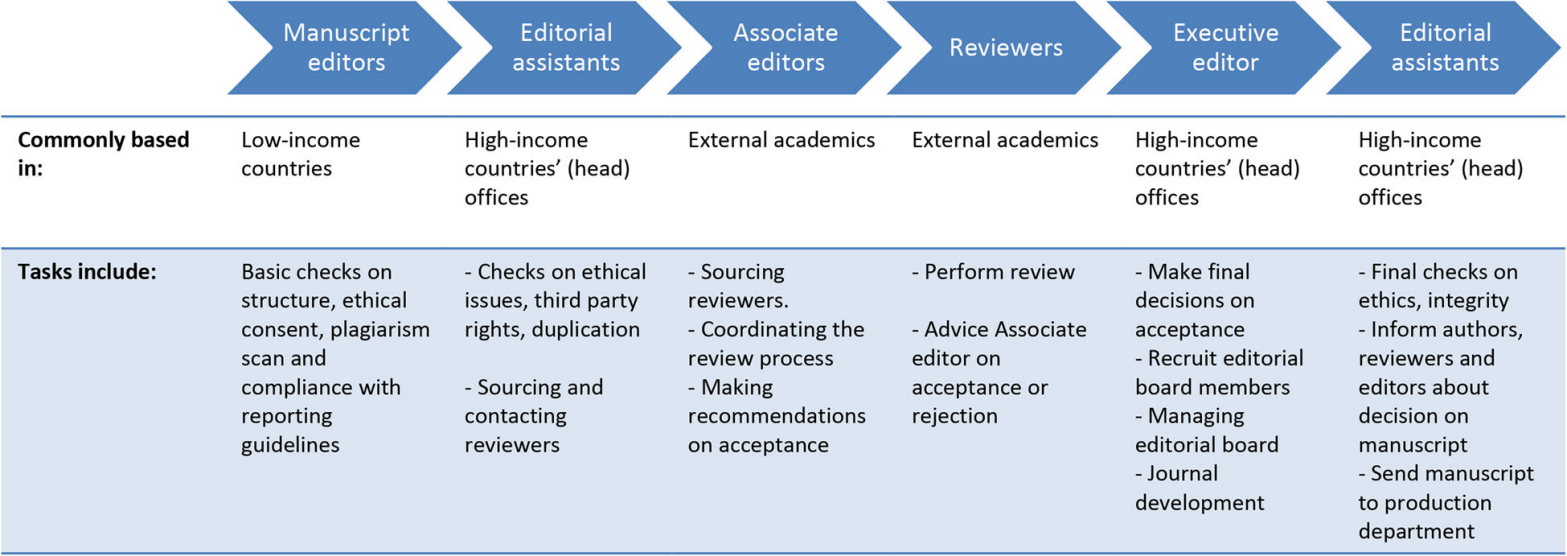
Schematic overview of the editorial process

When a manuscript is submitted through the online editorial management system, it will be handled by various actors, each with their own highly differentiated task:

- The first stage of manuscript processing is typically handled by people usually referred to as *manuscript editors.* Commonly, those editors are based in low-income countries. They might either be employed by the publisher or work for a vendor company. Manuscript editors perform basic checks on, for example, the manuscript’s structure, the plagiarism scan, declarations of ethical consent or issues, or compliance with reporting guidelines. They flag potential issues to the assistant editor and may send a bundled query to the author (the latter can typically be done only once in order not to slow down the process). Manuscript editors frequently communicate with assistant editors, the next chain of the process. As with much of the communication between different members of the publisher, this communication usually occurs via email or the online editorial management system, complemented by regular calls or meetings. Our information on these actors was obtained through talking with assistant editors and reading their written interaction with manuscript editors.
- The assistant editors or ‘editorial assistants’ (usually based in high-income countries’ head offices) consult the input from the manuscript editors and can perform some additional checks, for instance assessing compliance with ethical standards, third party rights, and duplication or overlap with other manuscripts. They might also send out queries to the author. In general, they try to balance several concerns: trying not to slow down the submission process, but also aiming for completeness and clarity before peer review. This was a common theme that was raised several times i.e. the importance of making necessary changes or requiring additional information so as not to inconvenience the peer reviewers, safeguarding them from instances of unclarity, poor language, or with manuscript structure issues. At publisher A, the assistant editors felt checking for ethical issues is the most important part of their job and maintains the company’s reputation: “We are very careful. We have to be very careful in order to protect the reputation of the publisher.” However, this also has a more formal side to it: “We make sure we’re not getting sued” (assistant editor).
  The assistant editors have clear targets with a number of manuscripts they are expected to handle per day. Handling a single manuscript is a highly standardised practice, which takes them between five and 25 min (depending on the level of experience of the individual and the complexity of the manuscript; most editors reported needing on average about 10 min per manuscript).
  The assistant editor then asks an associate editor whether s/he wants to handle the manuscript through the actual review process, usually by email or through the editorial management system. In some cases, the assistant editor might be in charge of sourcing and inviting reviewers, for which they will commonly consult the publisher’s database, or external databases such as Scopus, Web of Science, or PubMed. Commonly, they will resort to keyword searches to find reviewers, while keeping track of the number of papers an academic has recently reviewed and how reviewers were rated by associate editors in the past.
- The associate editors or editorial board members (who usually are external academics, i.e. not employed by the publisher, and sometimes called ‘academic editors’) receive the comments from the assistant editor. They source reviewers, when this is not already done, usually through their personal networks. In addition, they coordinate the review process and make a recommendation about acceptance/rejection to the executive editor. This work may be done on a voluntary, unpaid basis or editors may receive an honorarium. While some in the publishers’ offices argue these external editors are enrolled because of their expertise and connection with the research field and the community, others also point to a cost argument. They argue that publishers aim to have as many manuscripts handled by associate editors as possible: “ideally, all manuscripts are handled by external editors” (executive editor), for obvious reasons of lowering workloads for internal editors and hence reducing costs. This makes recruitment of external editors “an important part of journal development” done by executive editors (executive editor).
- The recommendations for acceptance or rejection by reviewers as well as the associate editor are then passed on to the executive editor, or editor-in-chief, who makes the decision. For the publishers we visited, these editors are usually employed by the publishers and were hence part of the editorial offices we visited. Executive editors may also have several additional roles: (i) if no external editor can be found, they take up editorial tasks of sourcing reviewers and managing the review process; (ii) they recruit new associate editors for the editorial board; (iii) they keep in contact with / manage the editorial board; (iv) they undertake journal development (redefining the journal’s scope, introducing new sections, managing editorial processes, marketing, etc.), and (v) they commission specific content for the journal (reviews, commentaries, thematic issues, etc.).
- The executive editor then reports the decision back to the assistant editor, who communicates the decision to the author. In the case that the manuscript is accepted, some final checks (similar to the initial checks after submission, but now stricter) are carried out by the assistant editor. This second round of checks is required as initial checks are only brief and relatively loose to prevent delays. After some potential final revisions, the manuscript is sent to the production units, which may be located in other countries, and which are tasked with typesetting and proofing. The various steps of the editorial process explained above are schematically depicted in Fig. 1.

We note that many of the processes described above apply to the entire portfolio of journals owned by the publisher. Practices and procedures are standardised across this portfolio and little fundamental distinction is made based on characteristics of journals, e.g. number of submissions or research discipline.

#### Journals owned by professional or learned societies

Besides many journals owned and operated by the publishers themselves, both publishers have a number of journals in their portfolio that are owned by professional or learned societies. For these journals the publisher might take on slightly different roles. Most commonly, the publisher and society negotiate a division of the tasks mentioned above, in which the publisher performs a subset of the tasks, while others are maintained by the society. Within such societies, the task differentiation is commonly far less pronounced than in the large publishers: usually many of the different tasks mentioned above are carried out by only one or very few people.

The same holds true for the independent journals of which we interviewed the editors. At their journals, the many different layers described above are commonly merged into three: a managing editor or editorial assistant performing the tasks of the above described manuscript editors and editorial assistants; an (external) handling editor resembling the role of the associate editor; and an editor-in-chief, akin to the executive editor within the publisher’s structure.

#### Publishers’ management of journals and editors

In addition to individuals in roles directly related to the handling of manuscripts, several other people are involved in the core business of daily journal management (i.e. apart from technical, maintenance, catering or safety processes).

- Managing editors or team leaders typically manage a group of assistant editors. They keep track of their performances (in terms of targets, which usually centre on the number of published/checked manuscripts), are an ‘escalation point’ for (difficult) issues with manuscripts, they provide training to new members, and they distribute new projects over the team members / journals. They might also have a say in decisions on the journals policy or peer review model.
- Journal managers manage the entire editorial process for a series or portfolio of journals. This person could be accountable for the process management of the journal, managing the editorial and peer review process, as well as for strategic management of the journal. This could include ensuring that journals are maintaining certain standards, for instance with respect to growth, author service, publishing integrity, etc. They can have a prominent role to play in journal policy and might be in contact with, and undertake negotiations with members of societies affiliated with the publisher.
- There are often various support teams, such as those that may help source reviewers in case a manuscript needs to be handled in-house or an associate editor cannot find reviewers. They do this based on bibliometric techniques, such as by using keyword searches, and use databases of academics doing reviews for similar journals. Another support team include for example those that manage editorial inboxes, by taking care of all author queries related to manuscripts ‘under review’. Also, the publishers now have dedicated support teams for research integrity related issues.

Additionally, several other layers of managerial roles complement the daily management of journals. These higher managerial layers are not, or less, concerned with daily operations, but rather focus on long-term strategies and more comprehensive projects involving large numbers of journals. Among these roles are chief publishing directors, product owners, and open access managers.

#### The external review process

The review process in the publishers’ journals is conducted pre-publication, mainly through either single-blind review (in which the reviewer knows the author’s identity, but not vice versa), or open review (in which both authors and reviewers know each other’s identity – in this case review reports may also be published online along with the published article). Some journals also uses the double-blind format (in which neither reviewers nor authors know each other’s identity). Text similarity scanners are used, but no other (semi-)automated digital tools (such as statistics or image manipulation scanners). Reviewers, who are selected by the editors, do not get the opportunity to communicate with each other in the review process. Some journals at both publishers now also offer the Registered Reports model for doing review [30]. However, editors report that the uptake of this model is still low and hardly any manuscripts are reviewed in this way.

#### Analysis of the work of publishers

In our assessment, the publication process at the big publishers is a very layered and hierarchical practice, organising the editorial process in a long procedural chain, with highly specialised division of labour. The process depends heavily on connections between, and coordination of the individual actors’ practices. These consist of procedural solutions of providing and delivering information, but also of signalling potential issues that may need intervention of higher layers in the hierarchy. The material components of the practices, mainly embedded in the infrastructure of the electronic manuscript management system, play a key role in aligning and connecting the different tasks. This facilitates fast interaction between the different layers of the process and coordinates it by keeping track of a manuscript’s status and of internal and external actors involved. We see this also reflected in the editorial process of some journals not directly related to large publishers. One of the managing editors of such a journal mentioned: “We use a very old-fashioned online system. It is very basic, it cannot do much, but it works.” In such smaller editorial teams, with less specialised division of labour, the online management system is less required to align the different actors in the editorial process.

### Innovating and changing editorial processes

#### Getting ideas

Editorial teams learn about the editorial innovations currently suggested or tried out in academic publishing in various ways, including some channels of particular interest for academic publishers. Even though we could inform the publishers about some recently suggested peer review formats, the majority of models we were aware of were already known to the editorial teams through other channels.

Several of the ideas travel with people moving between publishers or units within the same publisher: “The idea for the project comes from [the new team leader], who was on [another journal] and now joined our team. He took the idea with him. He made people aware of the project” (editor team leader). With people switching jobs between publishers, not only knowledge concerning (new) editorial practices is exchanged, but also competences required to perform such practices, hence allowing practices to spread. Other ideas might reach the team via professional contacts, such as software developers or academics, e.g.: “I got to know the people who are involved in establishing the platform” (executive editor). Similarly, editorial board members might suggest new approaches, or they might indicate issues with the current approach, thereby triggering discussion about how to solve such issues. This is one of the main information routes for suggestions to publishers from their community and the editorial board keeps the journal in touch with the community. Another editor mentioned the value of social media: “The main source would be Twitter, actually”, referring to online discussions and fast circulation of ideas on these platforms. Another occasion for new procedures or practices occurs when “the electronic management systems that we are using, enables something new. That is a major breakthrough for us” (publisher). Such software innovations give publishers the opportunity to quite easily implement something new, but they also show that other publishers are working on similar initiatives, creating pressure to keep up with competitors. What we thus see is that personal networks of people working for the publisher are important, especially for those working close the community of authors and reviewers. However, also publishing technology can be a conduit for innovations and can be crucial to their introduction to work processes.

In this discussion it is important to distinguish between larger and smaller projects, the larger projects being those either rolled out over many journals or those requiring large investments. Smaller projects may find their way to the project team via the above mentioned formal and informal networks. In contrast, larger initiatives will come “from very high up” (team leader of editors), referring to the top management of the company. One of the team members initiating many of the large new projects explained that projects arrive at their desk mainly in two ways: either the head of an innovation department suggests new initiatives after consulting with technology companies, which demonstrate novel opportunities, or “management makes contractual deals with other companies.” This might include external partners, usually commercial providers of automated tools or review services, which can assist in review and collaborate through large deals. A journal portfolio’s publisher added that, for the larger innovations: “We will take the same approach as any other company might take, by looking at the market and seeing where gaps appear. […] We will look at spots where growth may be realised […] or where we see an opportunity” (Publisher, referring to market analyses carried out by a dedicated team within the company). Therefore, information channels do not just pertain to knowledge about academic benefits of innovations, but also about business economics, in particular for larger innovations.

#### Decision makers

The same factors mentioned earlier, the companies’ internal hierarchy and the distinction between smaller and larger projects, become evident when we observed who can decide to start, continue or terminate new initiatives. Commonly, when ideas reach a publisher’s management team, they will first be implemented on a small number of journals, during pilot phases, before deciding to roll them out over a wider set of journals. Publishers hence opt for rather gradual transformation of the editorial practices, containing risks and creating learning opportunities. Currently, several trials involving smaller or larger modifications to the publication process are executed in journals managed by the publishers. These include the introduction of new checks carried out by the assistant editors, or more extensive modifications, such as offering pre-print possibilities to authors; implementing some form of Registered Reports, in which research protocols or plans are reviewed before results or conclusions are known; and the introduction of variants of open peer review, in which peer review reports are published alongside the articles.

In the discussions about who can decide to implement innovations, the publisher’s internal hierarchy becomes very visible. Speaking to several members at the office, the phrase ‘people higher up’ was very common to describe where decision-making power lies: “These decisions come from many levels above me” (senior member of product development team). “But I am not the one making the decisions about this. That will happen higher up” (team leader of editors). This is mainly the case when decisions about larger projects have to be made: “The decisions about such large projects will be made on the very top level of the chief publishing director. In general, decisions about such large projects, enrolled [publisher]-wide, will be on this level. Smaller projects might be handled at a lower level. It depends on the potential impact that the project has, in terms of investment, required structural changes and potential of improvement” (publisher executive).

However, even in decisions about smaller projects, the hierarchical structure of large publishers’ organisation becomes evident: “[…] in this case it was the chief editor who decided it, but she had to get permission from her manager” (executive editor). Or similarly: “Management would then decide whether this is a good thing to do from a business perspective. If they decide to go for it, we would just do the implementation right away” (associate in policy team). In practice, decisions are made by managers (who manage portfolios of several dozen journals) and people above them in the hierarchy.

The situation is more nuanced for society journals, i.e. journals owned by professional or learned societies in which the publisher has a mainly supporting role. These journals are largely autonomous and the publishers have only limited involvement with their editorial policies and processes. Hence, decisions about how to organise the editorial and peer review process are largely made by these societies and their editorial managers, thereby creating additional layers next to the publisher’s internal hierarchy. Societies can frequently lead the way in particular community developments and be a source of inspiration for the publisher. Conversely, if decisions involve discussions over a longer time period it may be more quickly implemented in journals entirely owned by the publisher.

Summarising, at the publishers we analysed, the decision-making structure seems to show both clear patterns of hierarchy and layering of the different editorial practices. Larger projects travel top-down through the company, driven higher levels’ interpretations of what innovations mean for their concerns. What constitutes a convincing argument for change varies between different layers in the hierarchy. It is to these meanings and arguments for change that we will now turn our attention.

#### Analysis of arguments leading to innovations

Observing editorial transformation processes or asking about past transformations to over 20 editorial office members, we analysed reasons that convince decision makers to enrol or implement new initiatives. Not surprisingly, the commercial aspect of the publisher plays a crucial role in these decisions, with typical expressions such as: “At the end of the day we’re still a business.” However, on closer analysis, this commercial argument manifests itself in quite specific ways, related to the publishers’ business model and corporate strategy. It is not just *that* business interests play a role in innovation decisions, but *how* these business interests are understood.

First, the publishers seem to be particularly keen on protecting or strengthening their reputation, even at the expense of additional resources. This becomes clear in the following excerpt from the field notes, from a conversation with a support staff member about new initiatives to detect misconduct cases:

‘I bring up that, because there are only relatively few integrity cases, it might not be worth a huge investment. She responds that: “One case can bring a lot of reputation damage” explaining that even though they have a lot of papers published and only relatively few of those contain issues, these still can cause major harm to the reputation of the publisher. I reply that hence, even though they might need to set up systems that take a lot of resources and may only catch a few cases, this may still be worth it, because it may prevent reputational damage: “Yes, absolutely”, she confirms. “There needs to be a lot of trust in the system.”’

Another consideration for the publisher, that is related to commercial considerations, is how the publisher can add value for researchers. This usually comes in a drive to speed up the editorial process, improve handling of submissions, decreasing turnaround times etc. From conversations with a number of publishing colleagues - at all levels - it was clear that projects are introduced where the publisher believes they are doing the right thing by the community in upholding standards that the community supports and are not introducing changes or innovations that would not receive support from the community.

A second way in which the commercial argument manifests itself, is through a continuous push to balance a need to speed up the editorial process, decreasing turnaround times and thereby decreasing costs, within the quality standards of the editorial process and consideration of the research community’s needs. An executive editor responds to my question of what would be convincing arguments for her manager: “A lot of it will be time: we obviously don’t do things that increase our turnaround times. Other factors include concerns about whether it is not too labour intensive, and we balance time and benefits” (executive editor). A member of the product development team explains: “The biggest cost for the publisher is the editorial process. […] And we do not want to undo any of the efforts that we made via other means in speeding up the process.” She claims that “this [the increased cost due to more time-consuming editorial processes] will form the major barrier to enrol the project on a wider scale” (senior member of product development team).

A closely related third manifestation of the commercial argument concerns the number of submissions a journal expects. Short turnaround times are not only beneficial to reduced costs, they also indirectly lead to increased submissions and hence revenue potential: “Speeding up is required for authors, because they want quick turnaround times, and for the reputation of the journal” (executive editor). Another executive editor explains: “In general, when you want to convince people, you need to show that you have the backing of the field. […] In the end it all comes down to the number of submissions we get” (executive editor). Or a more direct claim: “We could never suggest anything to journals if it either makes the authors or the reviewers less likely to work with the journal” (Senior Manager, Publishing Team). Another senior staff member working alongside society owned journals, told me that: “We persuade people to get more and better content.” Referring directly to the business value and corporate strategy of the publisher by strengthening its reputation, an assistant editor team leader stated: “… we always think about how we can improve the turnaround time, since we may use that as a selling point.” However, this commercial logic has to be balanced with academic standards and this may involve measures that may conflict with individual researchers’ interests. As another staff member later commented: “We reject papers, we retract papers, we publish papers, and each of these might be an unhappy circumstance for an individual author or individual reviewer. But what we try to do is the right thing by the community, upholding the standards that the community supports (...).”

A fourth argument also refers to adding business value, through responding to the needs of researchers in their various roles as editors, authors and reviewers. Factors here involve costs of introducing a new project while maintaining appropriate legal considerations and the perceived benefits to all parties of making a change. This becomes clear in a conversation with a publishing associate in the publishing policy and strategy team:

SH: “So if I understand you correctly, there are two main pillars on which you base your decisions, being first of all the legal aspect: Are we allowed to do this? And secondly the commercial or business aspects: Can we actually make money out of this?”

Interviewee: “Yes, yes, that is true. But usually we are not directly looking at how much money we can make with it, because obviously many of these projects, such as the […] project, are not directly making money. Instead, we usually ask how much money we can afford to put into this. Because it will just cost us money to build the system, but if reviewers like it, it will make the reviewers happier and they might be willing to review for us another time.”

SH: “And that might then make the review process go faster and hence lead to a more cost-effective process?”

Interviewee: “Yes, exactly. That is how it works” (publishing associate in the publishing policy, development and strategy team).

Another indication of what arguments are convincing can be observed in the monitoring and evaluation indicators for new project pilot phases. Several members of the editorial board explained how they would measure: “Turnaround times, the number of reviewers engaging, and the rate of reviewers accepting to review” (publisher executive), and: “The usual thing we would measure is the uptake by authors” [i.e. the number of authors opting for a newly offered service] (member of product team). Such performance indicators closely articulate the business concerns in operational terms and translate them into terms directly relevant to editorial innovations. As we noted earlier, these feedback and monitoring mechanisms are crucial mechanisms to maintain or transform practices. Specifying monitoring criteria that operationalise speed and uptake will increase the endurance likelihood of practices that align with business strategy.

This attempt to increase efficiency might have further consequences for specific scientific disciplines. As we noted earlier, there is an increasing desire to standardise editorial processes across journals within the same publisher, mainly driven by efficiency and economy-of-scale considerations. In this effort towards more standardisation, approaches are “commonly aligned with those of the largest set of journals that already use them or in which they naturally fit” (editor-in-chief). Hence approaches are typically modelled on disciplines in which the publisher holds most journals. This may decrease diversity in review procedures, potentially at the expense of approaches more suitable in other, smaller research fields in which the publisher holds only few journals.

However, commercial arguments are not the sole factor facilitating or hindering innovation. In the complex hierarchy of tasks within the publisher, a single group of people is particularly involved with the academic aspects of the editorial process. This group, consisting of executive editors or editors-in-chief, is fairly distanced from direct financial and business considerations. It is this group that seems to be particularly keen on improving research and for whom a publisher is clearly distinct from ‘any other company’. One of them claimed: “We are a company, but we are not a manuscript accepting machine. […] I really want to do it well.” He was positive about his colleague editors thinking about it the same way. “It needs to go well” he claimed, “else I do not want to work for the journal any longer” (executive editor). Other members of the company acknowledged this role, describing the executive editors as “the guardians of quality” (in-house editor).

Once again, we can hence observe how the meanings attached to editorial practices vary between different levels of the organisation. Whereas at the executive level, the practices are instrumental in facilitating company growth, fostering reputation or creating unique selling points, editors attach more academically informed meanings to the process, aiming to improve science and dissemination of scientific results. It is in the interplay of those layers and meanings that decisions about transformations of the editorial process are made.

#### Potential hurdles

Apart from analysing convincing arguments to implement or introduce new initiatives, the analysis of what constitute major hurdles to editorial transformations provides another interesting lens. Besides the legal restrictions mentioned above (such as privacy concerns or issues related to the General Data Protection Regulation), two main categories of impediments were foregrounded. These were explicitly articulated and summarised by one of our interviewees: “There are two main hurdles to innovation: People are very reluctant to change. In the end, we all want to keep things as they are. And there are the technical issues involved” (executive editor). Both impediments were expressed multiple times by members of the editorial team, which may indicate that either habits and conventions among certain actors, or the technical configuration of the electronic editorial system are indeed the main hurdles to innovation.

“Sometimes the reviewers have issues with that [new format of the review process], even though they accept to review in this way at the very start” (executive editor), implying that the instructions did not come across.

“I hope to see it being implemented soon. This will require some changes to the [name of the electronic editorial system]. That is always complicated. It will take a while” (team leader of assistant editors).

Especially the technical hurdle is closely related to the material aspects of editorial practices, whereas routines are tied to the competence aspects of practices. The preference to either fix the technology or improve the competences can depend on the level of configuration that is possible within a manuscript management system and the costs involved. It may also be constrained by whether technical change and “automated solutions” are considered preferable, because they require less behavioural change than individuals involved in the processes.

Hence some of the material and competence elements of (complexes of) practices, may provide barriers to transformation or innovation. As we discussed earlier, the infrastructural online manuscript management system plays a major role in connecting and coordinating the various editorial practices. However, while usually taken for granted and to some extent invisible, it gets foregrounded when transformations to the system have to be made. This aspect, common to, or even defining of, infrastructures [31], makes the system one of the major hurdles in innovating editorial practices. Interestingly, these infrastructural aspects are not fully determined by the publisher itself. Both the structure of the electronic manuscript management system, the developers of such systems, and the competences and habits of reviewers represent and originate in connections to the editorial practices at other journals or publishers, as well as the practices of grant review at funding institutes.

### Publishing in a changing landscape: open science and shifting expectations

Currently, several potentially fundamental changes are taking place in scientific publishing. These changes include a move towards open access publishing, increasingly demanded by major funding agencies [22], which has major impact on the publishers’ business models. They also include a growing discomfort of multiple stakeholders in research about the role of (commercial) academic publishers. Fuelled by discussions about large profit margins of such publishers [32], rising prices of subscriptions and open access fees, combined with simultaneous budget cuts in academic research, these objections have done considerable harm to publishers’ reputations.

In addition, the rise of other publishing formats, such as pre-print servers that no longer require the direct involvement of publishers [7], raises further issues about the role of publishers. People have started to ask questions about how publishers add value to the publishing process or the published literature and about the justification for spending large amounts of, mainly public, funds on publishing through large, commercial publishers [15, 33].

Unsurprisingly, members of the offices we visited were well aware of this changing landscape and the potential implications for their work and products. The awareness of the publishers’ need to demonstrate added value manifests itself in several ways. At one of the publishers, research integrity plays an important role in this. In a conversation with a senior team member, she explained that there is currently a lot of outrage against publishers:

“People say that we don’t need the publishers anymore, because they can just post research on their personal webpage or submit it to pre-print archives and have other people review it on these platforms. We therefore have to show the added value of publishing and our work [at the research integrity team] is a way of doing this.”

She expected that, by demonstrating an effort to uphold integrity and publishing ethics, publishers can increase trust in the work they publish. “It conveys a message that we are taking this seriously. It shows people that they can trust the work we publish and thereby we show the added value of publishers” (staff member).

In fact, such reputational considerations might even warrant deliberate financial losses: “Yes, I don’t think we are making any money on this journal. But it is our main open data sharing journal so we keep it because we want to seem like a publisher that supports open and transparent data” (member of product team). This is confirmed by a senior managing editor at the other large publisher: “there is no direct financial incentive for publishers to rock the boat to make a change. However, there is a long-term incentive to get involved. Publishers have to show that publishing is different from Wikipedia.”

This is in line with the dominant view on the changing status of publishers, which requires the publishers to take considerable action. Part of this action, driven by the shift towards more open access publishing, consists of a changing focus on ‘what the community wants’. While previously librarians would be the main source and spokesmen of ‘what the community wants’, publishers are quickly shifting their attention towards needs and desires of researchers, either in their role as authors or as reviewers, as exemplified in initiatives such as Publons, the use of the Journal Impact Factor to advertise to authors, publisher-facilitated preprint servers, or the appearance of mega-journals. This aligns with the publishers’ business needs: in the subscription-model, librarians were involved in deciding which subscriptions to buy, but in the open access model researchers themselves are more directly involved in deciding where to publish and hence where to spend money on publishing.

A stronger focus on transparency constitutes another trend among publishers that is fueled by external changes in the publishing landscape. By being more transparent about publishing work, for instance by showing how many reviewers had to be invited, publishers can demonstrate the effort that goes into the review process, thereby showing their added value: “We need to do a better job in showing how we have added value. Being open about review and the system is a way of doing this” (senior manager).

We thus see the specific meanings attached to parts of the editorial process. The practices of upholding research integrity and publishing ethics or increasing transparency might genuinely contribute to better research, but they simultaneously serve a direct business need. By several members of the publishers, they are understood in a fairly instrumentalist way, as mechanisms to safeguard or strengthen the publisher’s reputation.

In this respect, it is necessary to make a distinction between the meanings and values attached to specific practices by the publisher as a whole and by people in their individual capacities. In the conversations we had with employees we noticed a remarkable usage of the first person singular or plural. When referring to the company’s values and reputation, as well as their efforts to protect those, interviewees almost exclusively spoke in first person plural, e.g.: “We have to be very careful in order to protect the reputation of the publisher”, “We make sure we’re not getting sued” (assistant editor), and “we show the added value of publishers” (senior team leader). In contrast, when discussing more academic interest not as closely tied to the publisher’s commercial interest, interviewees were more common to speak in the first person singular: “I really want to do it well” (in-house editor) and “I dream about a world not so much focussed on indicators and turnaround times” (executive editor). This suggests subtle distinctions between personal or professional concerns and company concerns that attach a different meaning to innovations’ potential.

This coupling between individual and professional concerns was arguably stronger for the interviewed editors not working for large publishers. At least, no differentiation between first-person singular and plural was used in these interviews as described above for the publishers’ case. Nonetheless, the difference between commercial and editorial perspectives are still acknowledged, although now less sharply distinct: “We have an advantage, or maybe it’s a disadvantage, that we are not owned by a publisher, we are independent and non-for profit. We look at things from an editorial/scientific perspective rather than from a business perspective” (editor of independent journal).

In general, the editors of these more-or-less independent journals were also aware of the changing landscape that journals and publishers find themselves in. They likewise feel the need to respond to these changes, but perceive themselves as much more flexible to address these: “Many publishers only have 1 or 2 options. We are more flexible. We have more options to choose from and we can adapt quicker” (editor of independent journal).

## Conclusion

This study identified several factors that are important in transforming editorial practices of commercial publishers. To understand innovation considerations, we first note that the editorial process at large publishers is very hierarchically structured, with distinct tasks for distinct layers of the process and thereby a clear division of labour among these layers. Extensive training for in-house editors and elaborate guidelines and manuals maintain a highly standardised and routinized process. The many layers of the process clearly express the complexity and inter-relatedness of editorial practices, as a combination of many mundane, simple practices distributed over various people and places [19]. Thereby the study adds to the existing literature describing the rise of peer review as a strictly orchestrated practice, and the establishment of publishers and their organisation (e.g. [32, 34, 35]).

Analysing how editorial practices may be transformed, we conclude that, while information about new initiatives circulates widely, projects tend to be typically implemented only on a relatively small scale. For larger projects, managerial approval has to be obtained. Analysing the convincing arguments for management to make changes in editorial practices, we observe several recurring themes. The major factor encompasses a commercial interest, which is understood by these publishers as the importance to uphold reputation, shorten the editorial process and turnaround times, and increase willingness of researchers to be involved (either as editors, authors, or reviewers).

However, different meanings are at stake here, attached to editorial practices by people in different position at the publishers. Some assess and support editorial practices or innovations for how they might improve research. For others, the main question is what editorial processes mean for the publisher’s business model. Because the latter meaning is more common among people in higher layers of the companies’ hierarchy, this meaning tends to prevail in decisions on large-scale innovation projects. Hitherto, these commercial considerations have mainly been discussed concerning publishers’ attitude towards open access publishing or concerning predatory journals (e.g. [32, 36]). However, we show that they reach much deeper, influencing the core of the editorial process. The potential of editorial or peer review innovations is not just assessed in terms of whether they will improve research, but also in light of whether they strengthen the company. This potentially provides an important understanding not yet discussed in the extant literature on recent publishing practices, even though it is referred to in some historical work [2].

Last, factors commonly impeding rapid or large-scale changes in the editorial process are often related to infrastructural aspects such as the electronic editorial system or habits and conventions of individuals involved. These impeding factors, usually comprising the material and competence parts of editorial practices, are all examples of instances where the publisher’s editorial practices connect to those of other publishers, organisations or the wider publishing community.

## Discussion

This study relies on ethnographic data obtained through participant observations and in situ interviews with members of editorial offices. It is therefore affected by the inherent limitations of such methods [37]. In particular, the sensitive nature of the topics discussed, as well as the anonymity agreement with the actors involved, might cause some concern for the introduction of biases. We have aimed to overcome this by triangulating findings through obtaining information independently from multiple participants. In addition, as data were gathered locally, their generalisability to other publishers would remain to be tested. However, several participants, especially those that had a history of working at other publishers as well, indicated that practices and procedures at other publishers closely resemble those at the publishers studied. In this study, we were primarily interested in the (qualitative description of) the process of changing editorial practices. We hence did not focus on the frequency or number of such innovations, for which we can refer to previous work providing a quantitative assessment of editorial innovations at scholarly journals [8].

The debate about new initiatives to develop or improve the editorial system or peer review system is usually centred on academic arguments: how the review system might improve the research enterprise. Arguments about the advantages of open peer review or similar innovations highlight advantages for the research process, obtaining knowledge and distributing it. As new ideas about how to organise the editorial process emerge, academic or societal considerations are therefore predominant [5]. On a small scale, such considerations may well drive innovative projects in academic publishing. However, when it comes to large-scale implementation by commercial publishers, other considerations and motives come to the fore. We show that the editorial process is closely connected to commercial practices of creating business value, and the very specific terms in which business value is understood, such as reputation considerations and the urge to increase efficiency. This might help explain why some editorial innovations have currently been successfully implemented on a wide scale, such as plagiarism detection software, whereas others remain peripheral, in spite of strong arguments from their supporters. Arguably, those innovations aligning with the specific understanding of the publisher’s business model are most likely to witness successful implementation. This might provide valuable insights for future endeavours to innovate the academic peer review system and improve its functionalities.

## Data Availability

Transcripts of interviews and meetings during the field visits are stored on Radboud University’s internal server and are not accessible due to privacy and anonymity restrictions of participants.
